# Study of image sensors for enhanced face recognition at a distance in the Smart City context

**DOI:** 10.1038/s41598-023-40110-y

**Published:** 2023-09-07

**Authors:** José M. Llauradó, Francisco A. Pujol, David Tomás, Anna Visvizi, Mar Pujol

**Affiliations:** 1 Department of Computer Technology and Computation, University of Alicante, Alicante, Spain; 2 Institute of International Studies (ISM), SGH Warsaw School of Economics, Warsaw, Poland; 3 Effat College of Business, Effat University, Jeddah, Saudi Arabia; 4 Department of Computing Science and Artificial Intelligence, University of Alicante, Alicante, Spain

**Keywords:** Computer science, Information technology

## Abstract

Smart monitoring and surveillance systems have become one of the fundamental areas in the context of security applications in Smart Cities. In particular, video surveillance for Human Activity Recognition (HAR) applied to the recognition of potential offenders and to the detection and prevention of violent acts is a challenging task that is still undergoing. This paper presents a method based on deep learning for face recognition at a distance for security applications. Due to the absence of available datasets on face recognition at a distance, a methodology to generate a reliable dataset that relates the distance of the individuals from the camera, the focal length of the image sensors and the size in pixels of the target face is introduced. To generate the extended dataset, the Georgia Tech Face and Quality Dataset for Distance Faces databases were chosen. Our method is then tested and applied to a set of commercial image sensors for surveillance cameras using this dataset. The system achieves an average accuracy above 99% for several sensors and allows to calculate the maximum distance for a sensor to get the required accuracy in the recognition, which could be crucial in security applications in smart cities.

## Introduction

Facial recognition is an intensive research area and has become a critical tool in safety and security systems implemented at micro- (houses and apartments), mezzo- (industrial facilities, correctional institutions), and macro-levels (urban areas, etc.). Facial recognition systems are an integral part of Human Activity Recognition (HAR) applications. They include identity authentication, video monitoring, and surveillance in domains such as access authorization and control, traffic monitoring, and congestion management, and smart homes, among others. A great and increasing number of face recognition-based applications are used in public spaces, thus establishing themselves as a fundamental component of broader and interconnected systems geared toward ensuring safety and security in public spaces. In this context, (smart) cities around the world are becoming key venues where facial recognition applications are used as tools to support the safety and security processes of monitoring and surveilling public spaces^1–7^. Not surprisingly, the market of products linked to facial recognition technology is expected to grow to $9.6 billion by 2022^8^.

Advances in information and communication technology (ICT), and especially the groundbreaking progress in the field of artificial intelligence, including machine learning (ML) and deep learning (DL), translate into new opportunities in the related field of computer vision^9,10^. Indeed, over the past years, many recognition methods have been tested in different lighting environments, positional set-ups, etc. to realistically model the uncertainty inherent in facial recognition under real conditions^11,12^. Facial recognition with DL methods has achieved over 99% accuracy even when challenging, unconstrained datasets were used^13,14^. Several systems designed to recognize and prevent crime, including acts of violence, have been developed through live recordings of security cameras^15^, allegedly helping authorities prevent and address instances of risk and threat to safety and security^16^.

Regardless of these advances, which tend to report results obtained under the optimal conditions of the research laboratory or individual and random success stories, the situation on the ground is frequently more complex. Thus, the possibility of embracing Artificial Intelligence (AI) enhanced methods of facial recognition may be compromised. For instance, in many cases, the respective systems operate under very difficult data collection conditions. That is, surveillance cameras are rarely located/positioned in a way that would allow a clear view of the object being monitored. Often the object itself is too far from the camera to record quality images. In addition, atmospheric/weather conditions, or the availability of (day) light, may literally obscure the quality of the image recorded. Furthermore, it is not uncommon to find old, low-resolution cameras still in operation. Research^17,18^ that examines the application of facial recognition applications to identify perpetrators of criminal acts reports that the results are not sufficiently relevant. This is because either the cameras used in the experiments had a very low resolution or the computational requirements to achieve reliable results made their application in a real situation unfeasible. The same issues can be extended to face recognition at a distance^19,20^. Indeed, there is still a lack of reliable systems for facial recognition at a distance through security cameras, a field of utmost importance in the context of day-to-day operations of smart cities.

In summary, irrespective of revolutionary advances in ICT, with regard to monitoring and surveillance, due to the frequently poor quality of the input, the expected quality from the computer vision output is far less than optimal. In these circumstances, the full use of the possibilities AI – and especially computer vision – offer in the smarty city, remains limited. That is, regardless of the increasing accounts and success stories of how facial recognition applications may be used in the smart city space, accurate identification and recognition of facial features remain difficult. Moreover, as the debate on facial recognition techniques and applications unfolds, legal and ethical considerations render the utilization of facial recognition-based applications in public spaces increasingly contentious.

Taking the above observations as the point of departure, in this paper it is argued that an in-depth and thorough examination of specific aspects of facial recognition technology is necessary. One of these aspects is the recognition of facial features at a distance. The objective of this paper is to suggest ways of bypassing some of the limitations inherent in existing approaches and applications to facial features identification and recognition systems at a distance. To this end, the main contributions of this work are as follows:

- Due to the current lack of face datasets with images taken at a distance, a framework has been developed to create an extended dataset with faces at different distances from a regular dataset. As a result, a new dataset at different distances and for different image sensors can be obtained.
- Our proposal improves security through video surveillance in Smart Cities by:
  i. Any image sensor of a surveillance camera can be assessed in advance by estimating its accuracy for face recognition at a distance before it is installed in a real situation.
  ii. For a real application where cost may be a determining factor to purchase a set of cameras in different locations for local governments, enterprises, etc., our work allows us to optimize the trade-off between the best accuracy for a given distance and the camera cost.

The novelty of this work comes from providing a method for selecting an image sensor that effectively balances accuracy and cost for a specific distance. The paper thoroughly examines the performance of various image sensors in face recognition across different distances. The research findings show that enhancing the focal length of image sensors significantly improves accuracy, particularly at distances up to 20 meters, thus establishing their reliability for distant face recognition applications. This methodology facilitates pre-installation evaluation of surveillance camera image sensors in the context of a Smart City scenario.

The argument in this paper is structured as follows. The following section offers a brief overview of the ways in which facial recognition-based smart city monitoring and surveillance systems can be used to improve safety and security in the smart city. Emerging legal and ethical issues are highlighted. In the next section, the use of real optical sensors to capture facial features is discussed. The application of Machine Learning (ML) and Deep Learning (DL) techniques for facial recognition is elaborated. Comparison between the sensors to obtain which of them could be more useful nowadays with our method follows. The paper concludes with a discussion on the state-of-the-art of computer vision and how it behaves in the field of security.

## Materials and methods

### Smart sensors, face recognition at a distance and the smart city: an outline of the relevance of the problem

Facial recognition applications have proven useful in a variety of contexts, typically associated with the processes of monitoring and surveillance (global and reactive) of private and public areas. Monitoring tends to be interpreted as an activity that is situational, consistent with watching and checking a situation carefully for a period of time to discover something about it. Surveillance is a more contentious concept^21–23^, even if commonsensically interpreted as identical to monitoring. Surveillance is most closely associated with the continuous and systematic collection, analysis, and interpretation of data needed to design, implement, and evaluate certain strategies, policies, action plans, etc. In the context of safety and security, a well-designed surveillance system would integrate sophisticated AI-based tools and applications, including facial recognition-based tools, with technologies and techniques that enable data mining, data processing, and data analysis. Eventually, these should feed the management and decision-making process^24^. In addition, successive point-of-interest (POI) recommendation has become a hot research topic in augmented Intelligence of Things (AIoT)^25–27^, which can be of great importance in security applications in a near future.

Smart cities represent in this context one of the most interesting test-beds for monitoring and surveillance systems^7^. It is because, on the one hand, the integration of the built environment and the ICT infrastructure creates the basic conditions for the installation and utilization of devices necessary for the purposes of monitoring and surveillance. On the other hand, the density and velocity of social interaction in the urban space, including traffic, congestion, mobility, trade, etc., are a source of demand for a form of monitoring and surveillance. Monitoring and surveillance systems in use in the (smart) city space fulfill several objectives, including traffic lights control, traffic and congestion management (for instance during rush hours or emergencies), optimization of public transportation use, preempting risks to public safety (e.g. averting mob creation or mitigation of disease spread, like Covid-19). Increasingly, facial recognition-based tools are becoming a part of these systems, although they also raise serious questions related to the collection, storage, and use of personal data thus collected.

Supporters of the utilization of facial recognition-based tools in the (smart) city space tend to argue that solutions of this kind are convenient, while at the same time allowing the detection of suspicious (or irregular) behavior, identification of perpetrators sought by the authorities, etc. As always, the question is who and on which grounds has the right to collect the data, who has the right to use the data and for which purposes, how safe storage of the data is ensured, and so on. Researchers involved in this field of research are aware of the inherent risks of facial recognition schemes applied and used in China^28,29^, not only on the grounds of bias inherent in AI/ML in connection with the facial recognition and interpretation process. Certainly, along with technological progress and the emergence of increasingly sophisticated technologies that can be applied in the fields of surveillance and monitoring, several contentious issues will arise^30,31^.

However, the legal and ethical challenges hinted at in this section should not impede the research and discovery process. Consider the case of dual application technologies^32,33^. Consider, for instance, that smart monitoring and surveillance systems have the capacity of detecting dangerous situations caused by negligence, e.g. car accidents, or even preempting risks by identifying and setting alert should serious speed limit violations in the city space be taking place. In other words, in the quest for even more efficient and more accurate ways of navigating problems inherent in the already existing tools and applications, like in the case of the discussed here at distance facial recognition capacity, it is imperative that the ethical and legal considerations are taken into account. Only in this way can we design and implement technologies and tools that will serve society at large.

### Related work

The efficiency of face recognition algorithms is closely related to the quality of the input images. Consequently, this is especially challenging when working with low-quality images, which is also often the case in many applications of video surveillance. In relation to face recognition using security cameras, previous works have mainly focused on topics such as low resolution, facial recognition with infrared security cameras, or recognition at a distance.

Low-resolution face recognition is still a challenging problem. Since biometric systems have generally been trained with high-quality images, low-resolution input images are likely to result in errors in the recognition process. In recent years, there have been a significant number of works related to low-resolution face recognition that use deep learning techniques with Convolutional Neural Networks (CNN). Most of these works are based on image super-resolution^34^ and, generally speaking, there are two alternatives for the recognition process: (i) input low resolution images are compared with high resolution images in the dataset; (ii) both input and gallery images are low resolution face images. Therefore, Cheng et al.^35^ presented a new super-resolution deep learning method where joint learning of super-resolution and face recognition is introduced. Then, a two-stream CNN is first initialized to recognize high-resolution faces and low-resolution faces at the same time for video streaming applications^36^. Moreover, Yu et al.^37^ introduced two super-resolution networks with high accuracy for a single image super-resolution, in comparison to several previous works. In^38^ a residual learning deep neural network based gradient image super-resolution solution is developed. However, these deep learning face recognition models generally show a substantial degradation in performance when evaluated with native low-resolution face images and have other limitations, such as their difficulty to extract discriminative features from low-resolution faces and the trade-off between accuracy and computational cost.

Regarding facial recognition in video surveillance, recent works include^39^, where video surveillance applications are studied in depth, considering that low resolution can greatly affect the reliable recognition of individuals. An automatic pose invariant for Face Recognition At a Distance (FRAD) is presented in^40^, where a 3D reconstruction of faces is performed and Local Binary Patterns (LBP) are used for classification. As in many other areas, deep learning is also used for facial recognition in video surveillance (see^41–43^). These works have limitations in terms of their reliance on stereo pair images, limited consideration of other variations and there is no evaluation on large-scale datasets.

On the other hand, Wheeler^20^ stated that to train and test models, there is a lack of datasets focused on video surveillance and, moreover, it is common to use downsampling to simulate images captured at a distance. Therefore, Grgic et al.^17^ presented a face image database, the SCFace dataset, taken in an uncontrolled indoor environment using five commercial video surveillance cameras. The database has 130 different people and a total of 4,160 images with different qualities and at different distances. Although there are some other datasets presented in the last few years, they cannot be currently accessed due to recent changes in data privacy laws.

From these works it comes clear that face recognition at a distance poses several significant limitations. First, the resolution and quality of the captured images decrease with distance, leading to a reduction in the amount of critical facial information available for analysis. This reduction in resolution can be exacerbated in real-world scenarios where factors like poor lighting conditions, occlusions, and varying camera angles further hinder the process.

Secondly, the effectiveness of face recognition at a distance is highly dependent on the available hardware and imaging capabilities. Long-range surveillance cameras may struggle to capture clear and detailed facial images, which can impact the accuracy and reliability of recognition systems. Additionally, the computational demands of processing distant faces can be considerable, requiring specialized and powerful hardware, which might not always be feasible or cost-effective for widespread deployment. To overcome all the limitations presented in this section, ongoing research and development are necessary to improve the robustness, accuracy, and ethical implications of face recognition technology at a distance. Proper data collection, algorithm improvements, and the establishment of ethical guidelines are crucial steps in effectively addressing these challenges.

As a conclusion, facial recognition at a distance for security applications has mainly dealt with near-images captured by sensors that do not use leading edge technology. State-of-the-art surveillance cameras have very good resolution at close distances, but as the target person becomes increasingly distant, errors due to low-resolution face recognition may emerge. Consequently, our work aims to study the behavior of current image sensors when capturing images at long distances and to test how our face recognition algorithm is able to work with low-resolution images. In this way, a comparison of the accuracy of the recognition for each image sensor with respect to the distance of individuals from the lens will also be taken into account.

### Implementation

**Figure 1:**
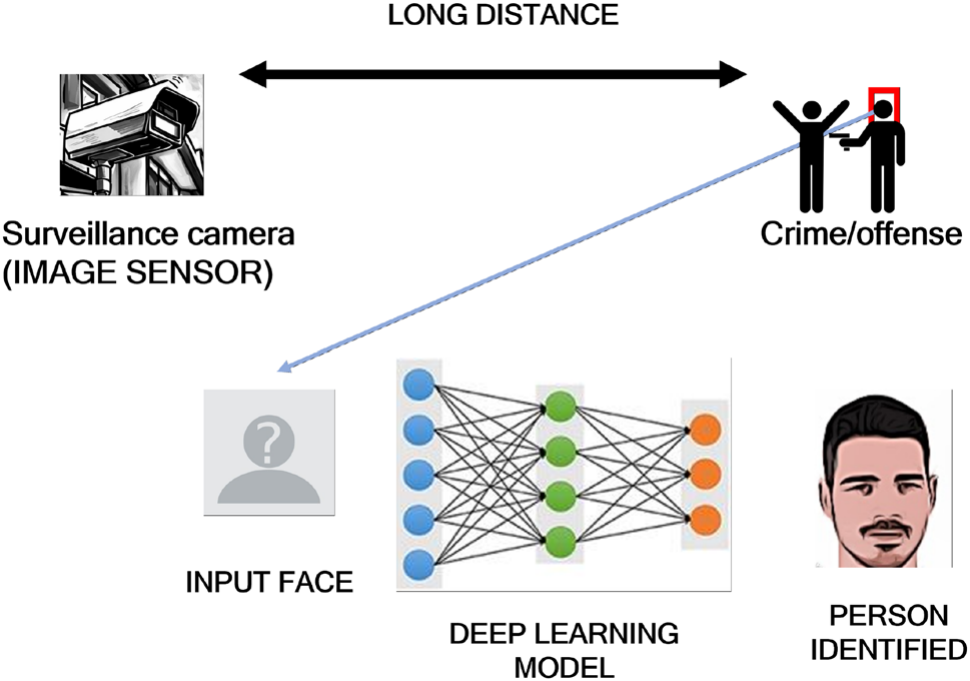
Graphical description of our proposal.

As mentioned in the previous sections, the main goal of this work is to extract faces from images taken by surveillance cameras, then use a deep learning model to recognize users in those images, and finally check the behavior of our system with a set of commercial image sensors at several distances. Figure 1 shows graphically how our system works.

#### Implementation of a facial recognition system at a distance

**Figure 2:**
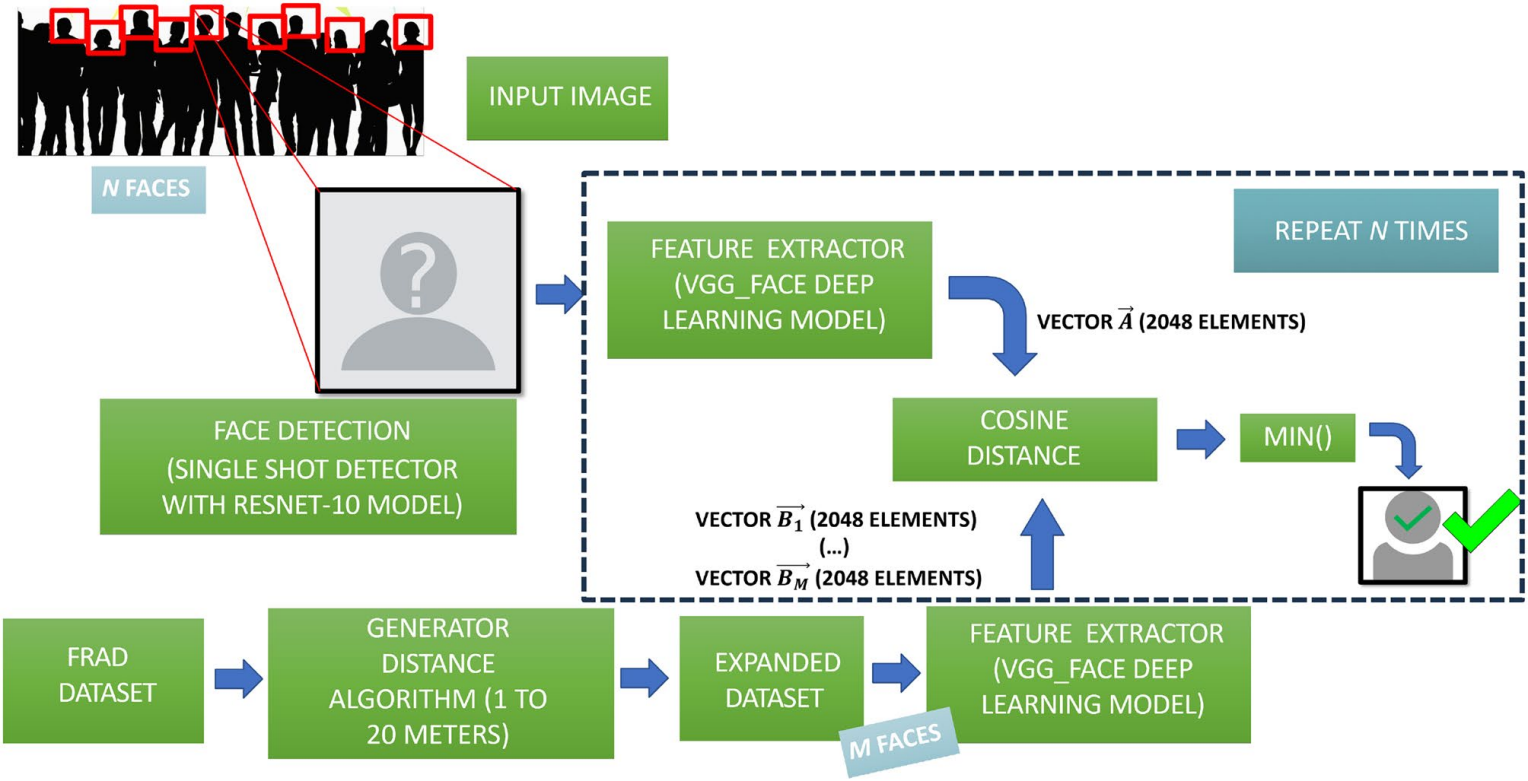
Architecture of our approach for facial recognition at a distance system.

A combination of Machine Learning and Deep Learning techniques, using Python^44^ as the programming language, has been taken into account to develop our method. Transfer learning^45^ techniques have also been used to optimize results and save training time. The overall architecture of our approach is shown in Fig. 2. The different steps followed in our model are explained below.

##### Face detection

For face detection, our model is based on the Single Shot Detector (SSD) framework with a reduced ResNet-10 model (for an extensive review of this model, see^46,47^), where the pre-trained model res10 ssd^48^ has been used. This model was created with the Deep Learning CAFFE library^49^ and gives robust results when identifying people’s faces at different distances and positions. In our case, it provides the position of the faces in the training and test datasets so that facial recognition can be performed afterwards.

##### Facial recognition

For facial recognition, once we have detected the position of the face, a feature extractor based on the VGG_Face model^50^ has been used, which was trained with the ImageNet database, obtaining 96.7% of success in face recognition in the near field. Let us consider that, in a general way, any input image *N* faces are detected, and that the training set will have *M* faces.

VGG_Face is one of the most popular and widely used facial recognition models. In this network, a feature vector of 2048 elements is taken from the last fully connected layer for each face image. Afterwards, the cosine distances^51^ between the feature vectors obtained from the faces detected in any input image to the system $\{A_1, \ldots , A_N\}$ and those from the training set of images $\{B_1, \ldots , B_M\}$ are calculated. The cosine similarity between two vectors $\vec {A}$ and $\vec {B}$, $\cos (\vec {A},\vec {B})$, can be defined as:

1 $$\begin{aligned} \cos (\vec {A},\vec {B}) = \frac{\vec {A}\cdot \vec {B}}{\Vert \vec {A} \Vert \Vert \vec {B} \Vert } \end{aligned}$$

The cosine distance $d_C$ between two vectors $\vec {A}$ and $\vec {B}$ is:

2 $$\begin{aligned} d_C= 1-\cos (\vec {A},\vec {B}) \end{aligned}$$

Then, a One-shot learning technique^52^ is applied. This technique is a classification task where one example (or a very small number of examples) is given for each class (an individual, in our case), that is used to train the model and make predictions about many unknown examples from the testing set.

The pseudo code for our proposed algorithm is shown in Algorithm 1.
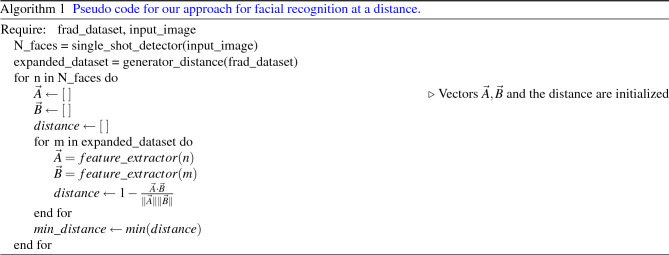


#### Generation of a dataset for FRAD

The lack of databases with images of faces taken at different distances has been reported in the literature^20^. Moreover, in the last few years some of the most popular datasets for face recognition at a distance cannot be accessed due to data protection laws. For this reason, and to test the detection range of our face recognition system with different sensors, an extended dataset from a regular dataset has been generated^53,54^. The new dataset contains images of different individuals with high resolution.

The generation of the dataset had to be realistic, since an image sensor does not collect the same visual information of a person at a particular distance as a smaller sensor at the same distance. Thus, the size of an individual’s face in an image taken by a surveillance camera in an urban area, where several other individuals may appear, will be very small the further away the target person is. As that person gets closer to the camera, his/her face will start to cover bigger areas of the image. Moreover, if the camera sensor has more resolution, that is, if it is able to take more pixels per image, a person’s face at a certain distance will use more pixels than the same face at the same distance, but captured by a camera with a smaller sensor.

Consequently, having an estimate of the size in pixels that a human face will occupy at a given distance using a particular optical sensor would be extremely useful. In our case, once this value is calculated, an extended dataset from the original high resolution dataset will be generated for that value by applying an antialiasing filter to downsample each original image to obtain the new dataset^55–57^. As a result, a new dataset at different distances and for different image sensors can be obtained.

To calculate the size in pixels of a human face in an image when the individual is at a distance from the camera, let us consider the relationship between the focal length in an optical system *DF*^58^, the distance from the object to the sensor itself *d* and the size of the sensor. In addition, the form factor of the captured image is considered to maintain the aspect ratio of the image. As a result, the distance can be calculated as:

3 $$\begin{aligned} d=\frac{D F \cdot H_R \cdot H_V}{H_P \cdot H_S} \end{aligned}$$

**Figure 3:**
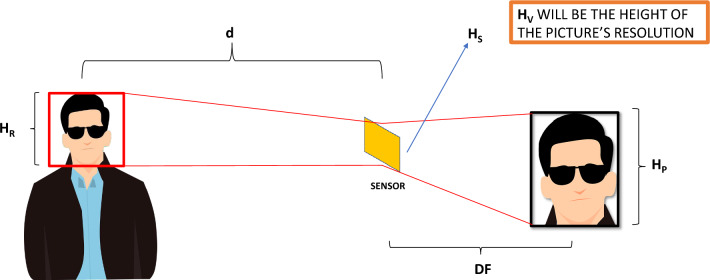
Graphical interpretation of the relation between focal length, face distance to the sensor and size of the image sensor.

In (3) *DF* is the focal length in mm, $H_R$ is the actual height of the object (that is, the human face), $H_V$ is the vertical height in pixels of the image, $H_P$ is the height in pixels that the object should have at distance *d* and $H_S$ is the physical height of the sensor in mm. See Fig. 3 for a graphical interpretation of Equation (3). An approximate size of 216 mm has been given to $H_R$, since previous works stated that this value can be considered as the average head size for an adult person^59,60^.

As a result, this approach allows us to synthetically expand the samples in a dataset to perform a study of which sensors behave better at certain distances from an object (face, in this case) recognition scheme. Some previous studies^61,62^ have shown that when working with synthetically created low-resolution images, the results can be slightly better than when using real low-resolution images. This small improvement in the recognition accuracy is considered acceptable, mainly due to the fact that (i) there is no dataset to address the problem presented in this article and (ii) regardless of the sensor used, this improvement acts as an offset on the sensors, as shown in previous works. Consequently, the results obtained in the next section are valid and can be extended to real datasets with slight changes in the accuracy results.

## Results and discussion

### Experimental setup

#### Description of sensors

As mentioned above, the main objective of this work is to conduct a study on the performance of various image sensors when used for face recognition at different distances. To this end, the experimental process will consist of testing the performance of nine different commercial sensors at a range of distances between 1 and 20 meters. The description of the sensors considered is shown in Table 1.

**Table 1:** Characteristics of the sensors.

Sensor ID	Model	Resolution (px)	Pixel size (μm)	Mpixels
S1	ICX098AL	640 x 480	5.0	0.3
S2	IMX462LQR/ LQR1	1920 x 1080	2.9	2.07
S3	IMX335LLN/ LQN	2592 x 1944	3.76	5.04
S4	IMX464LQR/ LQR1	2688 x 1520	2.9	4.09
S5	IMX533CQK-D	3015 x 3080	3.76	9.29
S6	IMX415-AAQR/ AAMR	3840 x 2160	1.45	8.29
S7	IMX412-AACK	4056 x 3040	1.55	12.33
S8	IMX571BQR-J	6280 x 4264	3.76	26.78
S9	IMX455AQK-K	9568 x 6380	3.76	61.04

#### Selection of datasets

**Figure 4:**
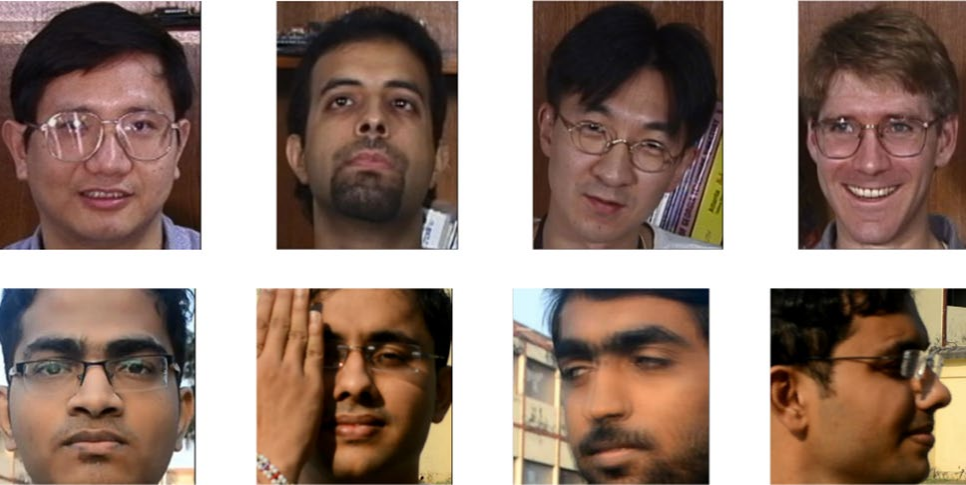
Example of images in the datasets. First row: Faces detected from the Georgia Tech Face database. Second row: Images from the QDF dataset.

Due to data privacy laws, there is a lack of public datasets that meet the requirements of the experiments in our research. Two databases have been used to perform the tests:

- The Georgia Tech Face database^63^. This dataset consists of a series of images of 50 people. The images were taken in different sessions on separate days in an indoor environment with controlled lighting and different poses for each individual were considered. For each subject, a total of 15 photographs were taken, which is a total of 750 images. These images were taken at a distance of near-field (0.5 to 1 m) and with a sensor resolution of 640 × 480 pixels. The average size of the faces in these images is 150 × 150 pixels. See Fig. 4 for some examples of images in this dataset.
- The Quality Dataset for Distance Faces (QDF) dataset^64^ contains naturally deteriorated facial images captured at various distances and with varying visual quality. The database contains images of 100 subjects with 32 variants with respect to distance, pose, partial occlusion, and illumination, making a total of 3200 images. A Nikon D-5200 camera was used to capture images and videos with a resolution of up to 1920 × 1080 pixels. Some images in this dataset are shown in Fig. 4.

All experiments were performed in accordance with relevant guidelines and regulations.

**Figure 5:**
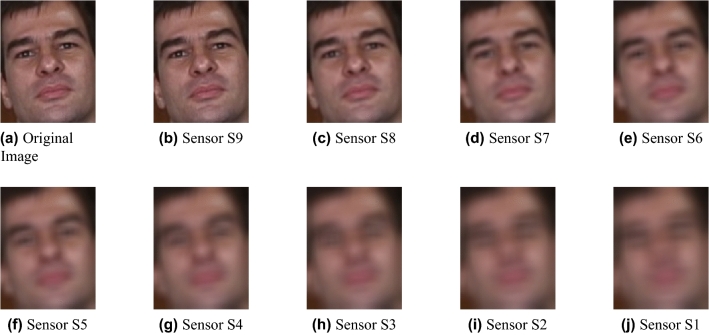
Example of synthetic images from the Georgia Tech Face database for each sensor at a distance of 5 meters.

**Figure 6:**
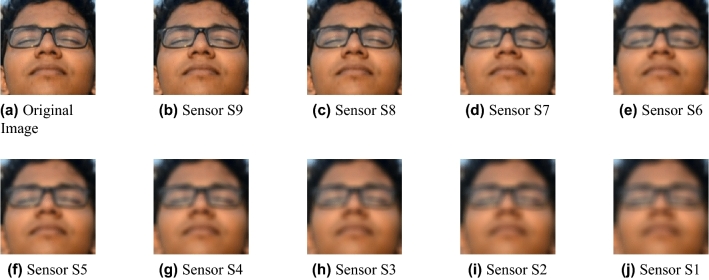
Example of synthetic images from the QDF dataset for each sensor at a distance of 5 meters.

As a result, we have used both databases to generate a new extended dataset that meets the requirements of our problem. All the images in the Georgia Tech Face database and QDF dataset were taken in daylight conditions. We randomly selected 15 subjects from the original Georgia Tech Face dataset and 15 subjects from the QDF dataset, considering for the QDF database only images in a near field (i.e. distance = 1 m). As a result, as images from the original datasets are in the near-field, the new dataset does not need any scaling or sampling for better visualization of small images. Then, the process explained in Section Implementation is used to create the synthetic images at different distances, ranging from 1 to 20 meters in steps of 1 meter. Thus, for each sensor at a specific focal length, we obtained 300 images for each distance, adding up to 9000 images to train and test the models. Considering as a distance 5 meters, some examples of the synthetic images generated for both datasets from all nine sensors are shown in Figs. 5 and 6.

For facial detection, the pre-trained model res10 SSD^48^ has been used, as shown above. To train the network, the model has been adapted to detect only one face per image since this is the case of the images in both databases. This way, the detection is optimized.

To carry out the evaluation, leave-one-out cross-validation was used for each distance in the range 1 to 20 meters: from the 300 reference images for each distance, in each iteration, 299 were used for training and 1 for testing. The final result for each distance is the average of these 300 evaluations.

Finally, it must be pointed out that the novelty of this work lies in its provision of a method that enables the effective balance of accuracy and cost in the selection of an image sensor for a specific distance, as it will be shown in the next section.

### Results

#### Sensors accuracy

Face recognition has been performed using the scheme discussed in Section Experimental Results. To do this, the distance is in the range of 1 to 20 meters, with increments of 1 meter. The VGG_Face Convolutional Neural Network (CNN) descriptor has been used to extract features and then to provide these features to six popular and robust classifiers when assigning a new input image to the corresponding class: Decision Trees, k-Nearest Neighbors (k-NN), Naïve Bayes, Multilayer Perceptron, One-Shot and Support Vector Machines (SVM).

Table 2 shows the average accuracy for every sensor with different algorithms in a short distance (1 to 20 meters). The focal length was set at 3.6 mm for all sensors. The row *Average* shows the average performance of each algorithm for the sensors studied. The bold font represents the highest recognition accuracy for each sensor. The best performing algorithm is SVM, closely followed by One-Shot.

**Table 2:** Average accuracy of algorithms’ performance.

Sensor ID	Decision		Naïve	Multilayer		
	Trees	k-NN	Bayes	Perceptron	One-Shot	SVM
S1	0.2160	0.3678	0.3700	0.4408	0.4687	**0.4853**
S2	0.3565	0.6192	0.6055	0.7143	**0.7575**	0.7492
S3	0.4985	0.8692	0.8165	0.9142	**0.9465**	0.9438
S4	0.3565	0.6192	0.6055	0.7143	**0.7575**	0.7492
S5	0.2825	0.4892	0.4845	0.5700	0.6080	**0.6120**
S6	0.6388	0.9877	0.9495	0.9887	0.9970	**0.9973**
S7	0.6102	0.9788	0.9257	0.9795	**0.9932**	0.9928
S8	0.2825	0.4892	0.4845	0.5700	0.6080	**0.6120**
S9	0.2808	0.4895	0.4855	0.5703	0.6077	**0.6122**
**Average**	0.3914	0.6566	0.6364	0.7180	0.7493	**0.7504**

The highest accuracy for each sensor with the considered algorithms is in bold.

The results obtained after analyzing the recognition accuracy of each of the sensors show that sensors with a higher number of Mpixels (*S8* and *S9*) do not necessarily achieve a higher recognition rate. This result reflects that also the pixel size strongly influences the final performance of the sensor, since both the number of MPixels and the pixel size are correlated with the actual height of the sensor ($H_S$). As stated in Equation (3), the height in pixels of the face ($H_P$) decreases as $H_S$ increases. Thus, sensors with a balanced pixel size and number of Mpixels perform better in this task. This is the case of *S6*, with a pixel size of 1.45 μm and 8.29 M pixels, outperforming *S9*, with 3.76 μm and 61.04 Mpixels.

#### Results using support vector machines (SVM)

**Figure 7:**
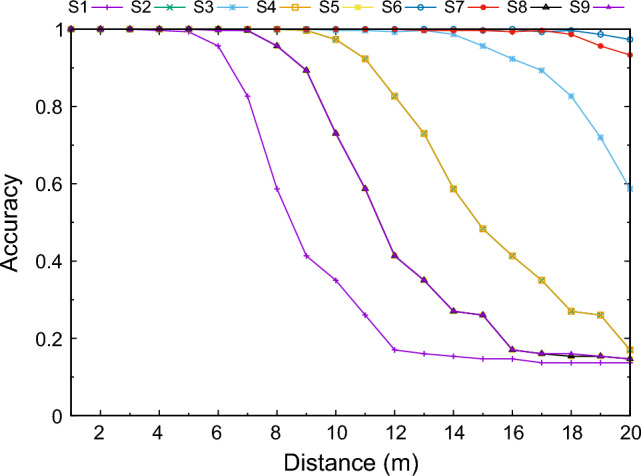
Results for each sensor depending on the distance using SVM.

Since SVM is the classifier that behaves better in the recognition process, Fig. 7 shows the performance of each sensor for this classifier depending on the distance.

From this figure, it can be seen that only the sensors *S6* and *S7* achieve a performance of more than 90% for all distances in the proposed range. We can also see that at short distances (up to 5 meters, approximately) all sensors behave properly, since they obtain a recognition rate similar to the VGG_Face model (96.7%). *S2* and *S4* overlap in the results obtained, whereas *S8* and *S9* also obtained very similar results over the entire distance range.

To improve the quality of the recognition system of the other sensors, the focal length of the lens can be increased. To clarify this, Table 3 shows the focal length necessary to make each sensor achieve at least 90% performance in the proposed distances. The tests have been performed considering that each sensor is used to recognize users at the maximum distance (20 m). *S*6 and *S*7 are not included in this table, since they already obtained more than 90% accuracy using a focal length of 3.6 mm.

**Table 3:** Accuracy achieved by the sensors at the maximum distance (20 m) increasing the focal length, using SVM.

Sensor ID	5 mm	6 mm	8 mm	12 mm
S1	0.1533	0.1700	0.4133	**0.9567**
S2	0.5867	0.8267	**0.9967**	1.0000
S3	**0.9733**	0.9933	1.0000	1.0000
S4	0.5867	0.8267	**0.9967**	1.0000
S5	0.2600	0.4133	0.8933	**1.0000**
S8	0.2600	0.4133	0.8933	**1.0000**
S9	0.2600	0.4133	0.8933	**0.9967**

In bold, the first time the sensor achieved the 0.9 accuracy threshold.

From Table 3, it becomes clear that with a focal length of 12 mm, all sensors achieved an accuracy of more than 90%. The sensor *S3* is above this value with a focal length of 5 mm, *S2* and *S3* with 8 mm, and the rest with 12 mm.

#### Results on maximum distance calculation

**Figure 8:**
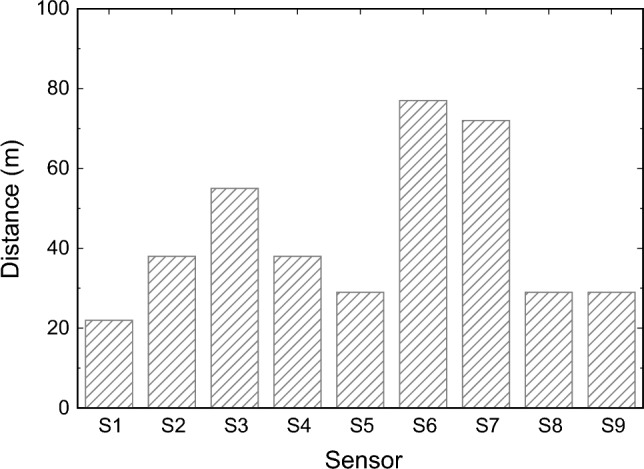
Maximum distance where each sensor was able to achieve more than 90% accuracy using a 12 mm focal length.

In the last experiment in this section, the goal was to calculate the maximum distance in which the sensors can achieve 90% accuracy using the 12 mm focal length. The results are shown in Fig. 8.

As expected, sensors *S6* and *S7* maintain the highest accuracy in face recognition at a very large distance, ranging between 75 to 80 meters in this case. Thus, both sensors could be tested in real situations, such as video security systems for Smart Cities, where potential offenders may be recognized from far away, to adapt the proposed face recognition scheme to achieve high accuracy in different real scenarios.

### Comparison with other works and discussion

Let us now compare our proposal with other related works. Table 4 shows a comparison of the best average results of the nine sensors considered in our system and the best results achieved in the following works; all of them have used the Georgia Tech Face Database:

- See et al. approach^65^, where a combination of a Gabor classifier with Random Forest was presented.
- Coşkun et al. proposal^66^, where a modified CNN architecture with some normalization operations and a Softmax classifier was used.
- William et al.^67^ introduced a face recognition scheme using FaceNet with two pre-trained models, CASIA-WebFace and VGGFace2.
- Zeghina et al.^68^, whose paper implements face recognition based on the Harris detector and CNNs.
- Horng et al. work^69^, where a super-resolution scheme with deep convolution neural networks is used to transform images from low-resolution to high-resolution.

**Table 4:** Comparison with other works.

Other methods	Accuracy
See et al.^65^	0.8920
Coşkun et al.^66^	0.9880
William et al.^67^	1.0000
Zeghina et al.^68^	0.9741
Horng et al.^69^	0.9500

Our proposal	Accuracy
S1	0.4853
S2	0.7575
S3	0.9465
S4	0.7575
S5	0.6120
S6	0.9973
S7	0.9932
S8	0.6120
S9	0.6122

The results in Table 4 show that our method achieves high accuracy and outperforms many of the other state-of-the-art works. The only work dealing with low-resolution images is^69^, whose best results are below ours (95 % vs 99.73% for the sensor *S6*). Then, William et al.^67^ obtained 100% accuracy for face recognition using the Georgia Tech Face database. However, their approach does not take into account face recognition at a distance. Therefore, it can be said that our proposal can be used as a reliable tool for face recognition at a distance, since (i) the image sensor of a surveillance camera can be evaluated prior to being installed in a real situation; (ii) the image sensor that optimizes the trade-off between the best accuracy for a given distance and camera cost can be selected using our proposal. Thus, it can make a successful contribution to improving security through video surveillance in the near future in smart cities.

## Conclusion

Smart cities serve as nodal points where the question of the use of monitoring and surveillance systems is supplied to provide a basic framework in which the safety and security of city inhabitants may be safeguarded. In other words, smart monitoring and surveillance systems have become one of the fundamental services that local authorities need to provide if the (smart) city is to operate in an efficient manner. Efficiency in this context is best defined by reference to safety, inclusion, resilience, and sustainability.

In this article, we have addressed the problem of facial recognition at a certain distance. A methodology has been introduced to generate a face dataset for a range of distances in case they are not available. In this way, nine commercial sensors have been considered and a dataset has been generated to meet the parameters of each sensor. The results of the recognition process show that some sensors achieve an accuracy greater than 99% for a range of distances between 1 to 20 meters, and even when the subject’s distance is between 75 to 80 meters, sensors *S*6 and *S*7 have an accuracy greater than 90%.

However, it is evident that these results come from a synthetic database and the images in this dataset have only one person per image and homogeneous background. As a future work, further tests with real video surveillance images, where more than one person appears per image and the backgrounds are heterogeneous, must be completed to verify the results obtained in this work. Despite this, we consider the results of this work to be promising and allow the selection of surveillance cameras with an image sensor that meets the requirements for any specific application.

## Data Availability

The Georgia Tech face database (http://www.anefian.com/research/face_reco.htm) which was taken in two or three sessions between 06/01/99 and 11/15/99 at the Center for Signal and Image Processing at Georgia Institute of Technology and is publicly available at http://www.anefian.com/research/gt_db.zip. The Quality Dataset for Distance Faces (QDF) dataset, which is available on request at https://sites.google.com/view/quality-based-distance-face-da/. The database contains images of 110 subjects with 32 variants with respect to distance, pose, partial occlusion and illumination and it was developed in Indian Institute of Technology (IIT) Kharagpur, India. As specified in the End User License Agreement (https://drive.google.com/file/d/10sU4aMr_fPoyk6x-a7TlU_LT_8Bn5SpS/view), we agreed with all the terms.No administrative permissions were required to access the raw data used in our study.
